# Integrated Plasma and Tumor Proteomics of Nasopharyngeal Carcinoma in a Moroccan Cohort

**DOI:** 10.3390/ijms26125771

**Published:** 2025-06-16

**Authors:** Ayman Reffai, Michelle Hori, Ravali Adusumilli, Abel Bermudez, Houssam Haddad, Nezha Tawfiq, Sharon Pitteri, Mohcine Bennani Mechita, Parag Mallick

**Affiliations:** 1 Canary Center for Cancer Early Detection, School of Medicine, Stanford University, Stanford, CA 94305, USA; aymanr@stanford.edu (A.R.);; 2 Intelligent Automation and BioMed Genomics Laboratory, Biology Department, Faculty of Sciences and Techniques of Tangier, Abdelmalek Essaadi University-Tetouan, Tangier 90000, Morocco; 3 Oncology Clinic of Tangier, Tangier 90000, Morocco; 4 Mohamed VI Center for Cancer Treatment, Ibn Rochd University Hospital (CHU), Casablanca 20100, Morocco

**Keywords:** nasopharyngeal carcinoma, proteomics, biomarkers, differentially expressed proteins, plasma, bioinformatics, molecular patterns

## Abstract

Nasopharyngeal carcinoma (NPC) is a multifactorial disease mainly affecting the Southeast Asian and North African populations. Critically, there is a dearth of available circulating biomarkers for NPC. Additionally, as of this writing, there have been no prior plasma proteomics studies on NPC in the Moroccan population. Accordingly, there has been no integrated analysis of tumor and plasma for NPC in the Moroccan sub-population. Label-free proteomics analysis was conducted on 25 samples of Moroccan origin (10 NPC samples and 15 healthy control samples). Each sample was depleted of albumin, fractionated into eight fractions, and then analyzed using Liquid Chromatography–Tandem Mass Spectrometry (LC-MS/MS). A total of 291 proteins and 2702 unique peptides were identified across all samples. In total, 16 proteins were differentially expressed (DEPs) between NPC cases and healthy individuals. Of these, three showed prognostic significance, while four demonstrated diagnostic potential. A pathway analysis showed significantly enriched terms related to the immune response and chronic inflammation, revealing acute-phase proteins as differentially expressed. The investigation of patients with early and advanced stages of NPC revealed two DEPs, while four additional DEPs were identified across the three defined clusters of NPC. Across all comparisons, DEPs, such as H2A, IGHG2, SERPINA3, SAA1, CRP, PIGR, and APOA2, have shown potential as biomarkers for NPC, with several being identified for the first time. We additionally compared the plasma proteomic profile of NPC with the tumor proteomic profile, highlighting that deeper proteomics analysis of plasma may be required to quantify additional putative biomarkers that may be shed from the tumor into the blood. Our research presents the first plasma proteomic profile of NPC in Morocco and North Africa, identifying proteins that might ultimately have diagnostic and prognostic potential.

## 1. Introduction

Nasopharyngeal carcinoma (NPC) is a distinct head and neck cancer characterized by its epidemiological patterns and molecular landscape. It arises from epithelial cells in the nasopharynx, which is the upper part of the pharynx located behind the nasal cavity. NPC mainly affects Southeast Asia, Southern China, and North Africa, including Morocco. The age-standardized incidence rates (ASRs) in these areas range between 2 and 10/100,000 annually [1]. In contrast, NPC is uncommon in other regions, such as North America and Europe, where ASRs are less than 0.5 per 100,000 people per year. In 2022, there were 120,434 new cases of NPC reported globally, with a mortality rate of 73,482 in both sexes and all age groups [1].

The histological types of NPC are categorized as keratinizing, non-keratinizing (differentiated and undifferentiated), and basaloid squamous cell carcinoma, based on the WHO histological classification (2022). Undifferentiated squamous cell carcinoma is the most prevalent histological subtype of NPC in endemic areas [2]. The latter has been strongly linked to infection by the Epstein–Barr Virus (EBV), one of the most studied risk factors for NPC, as well as a higher incidence of distant metastasis [3].

Established etiologic factors for NPC include an interaction of genetic, environmental, and viral factors, such as the EBV. In addition to genetic predisposition, somatic mutations are also significant contributors to the development and progression of NPC. Large-scale DNA sequencing studies have identified the mutational profile of NPC and the involvement of multiple signaling pathways, such as MAPK, NF-kB, and PI3K-AKT, in this complex disease [4,5]. While genomic and transcriptomic studies have provided significant insights into the genetic and molecular patterns of NPC, proteomics offers a complementary approach to understanding biological processes and signaling pathways in cancer by directly analyzing the functional molecules in cells (proteins). Since the start of the Human Proteome Project launched in 2010, proteomics has made incredible progress in uncovering molecular signatures and driving biomarker discovery [6].

Nasopharyngeal carcinoma is marked by a late diagnosis problem, with most patients being diagnosed at a late stage [7]. This delay heightens the risk of distant metastasis, resulting in a worse prognosis. Therefore, early detection is crucial for better clinical outcomes. Unfortunately, to date, there are no existing circulating biomarkers that may be used to diagnose NPC at its earliest and most treatable stage.

Despite recent progress, proteomics studies on nasopharyngeal carcinoma, particularly through plasma samples, remain very limited. Additionally, most studies have been conducted on patients from Southeast Asia and Southern China. Peng et al. (2024) [8] recently performed a proteomics analysis on small extracellular vesicles derived from plasma, identifying the CA1 protein as a potential biomarker for NPC diagnosis. Similarly, Zhang and colleagues (2022) [9] investigated plasma protein profiles to differentiate between responders and non-responders to induction chemotherapy (IC). Their study revealed four potential biomarkers for the early prediction of IC response in patients with locally advanced NPC [8,9]. To the best of our knowledge, this study marks the first investigation of the NPC plasma proteome in Morocco and across the African continent. Our goal was to characterize the proteomic profile of NPC by analyzing plasma samples from patients and healthy individuals in Morocco, a North African region with a high NPC prevalence. We additionally examined the relationship between the tumor and circulating proteomes of NPC.

## 2. Results

### 2.1. Protein Identification and Clustering

**Figure 1:**
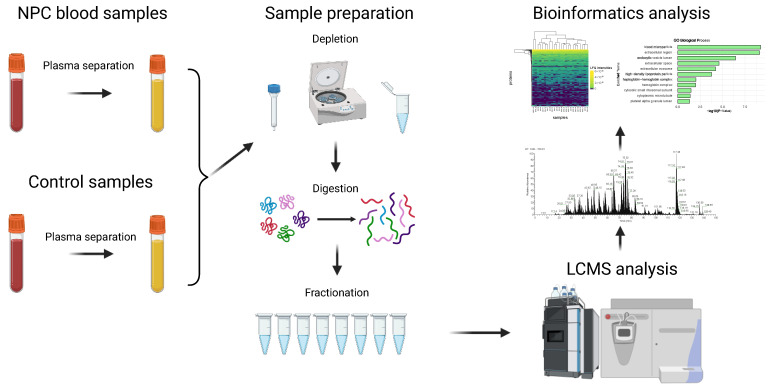
Label-free quantification shotgun proteomics workflow of plasma samples.

**Figure 2:**
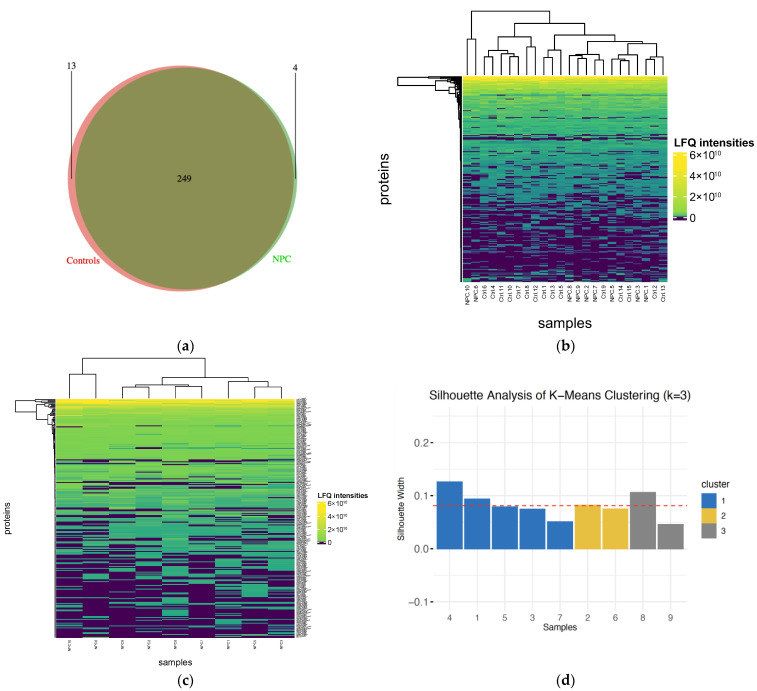
Protein identification and clustering across samples. (**a**) Venn diagram illustrating the identified and overlapping proteins between patients with NPC and healthy individuals; (**b**) heatmap showing the label-free quantification intensities of proteins in all samples; (**c**) heatmap of the label-free quantification protein abundances across all NPC samples; (**d**) silhouette plot representing the three identified clusters of NPC.

Label-free shotgun proteomics analysis was performed on all protein fractions (*n* = 200) from 25 plasma samples, including 10 NPC cases and 15 healthy controls, as presented in Figure 1. One of the NPC samples did not show consistent LC-MS results, so only nine NPC samples with eight fractions each, along with the control samples, were retained for the bioinformatics analysis. In total, 291 proteins without contaminations and reverse proteins were identified, along with 2702 unique peptides ranging from 7 to 45 amino acids (Supplementary Tables S1–S3 and File S1). The identified proteins in different groups were illustrated using a Venn diagram. A total of 253 proteins were found in patients with NPC, while 262 were detected in healthy controls, with 249 proteins overlapping between the two groups (Figure 2a). A heatmap was generated to visualize the distribution of proteins’ LFQ intensities in all samples (Figure 2b).

**Figure 3:**
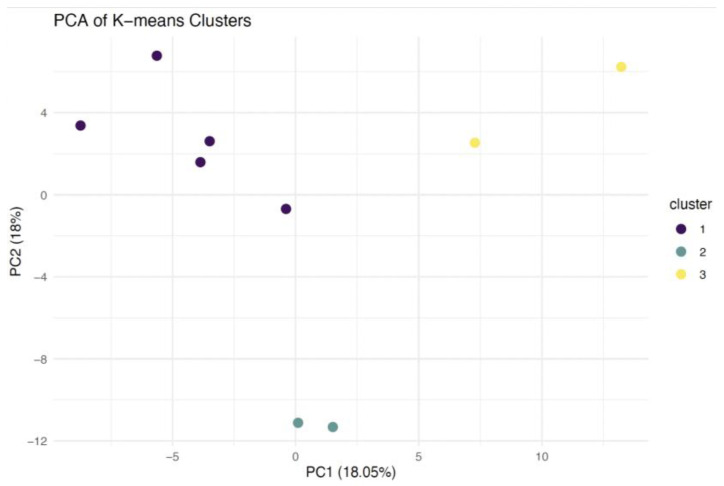
PCA 2D plot of identified clusters of NPC.

Furthermore, K-means clustering and silhouette analysis were performed across a range of k from 1 to 7 to examine the potential existence of interesting groups/clusters within NPC cases. This analysis revealed three defined clusters with the highest silhouette score of 0.081240. The first subtype contained five samples, while the second and third contained two samples each. A heatmap was used to present the distribution of proteins’ LFQ intensity across all NPC cases (Figure 2c). A silhouette plot was generated to represent the three clusters of NPC (Figure 2d). Principal component analysis (PCA) was also conducted to illustrate the identified groups in a 2D plot (Figure 3).

### 2.2. Differential Expression Analysis of NPC

**Figure 4:**
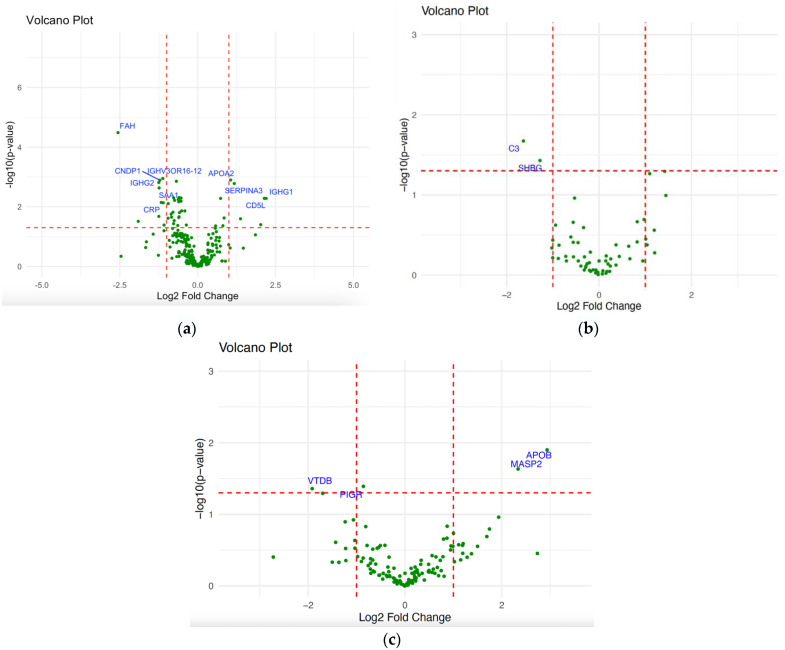
Volcano plot visualization of the differentially expressed proteins with an absolute 1 log2 fold change and *p*-value ≤ 0.05: (**a**) Patients with NPC versus healthy controls, (**b**) patients with early versus advanced stage of NPC, (**c**) NPC clusters condition (cluster 1 vs. 2, cluster 1 vs. 3, and cluster 2 vs. 3).

**Figure 5:**
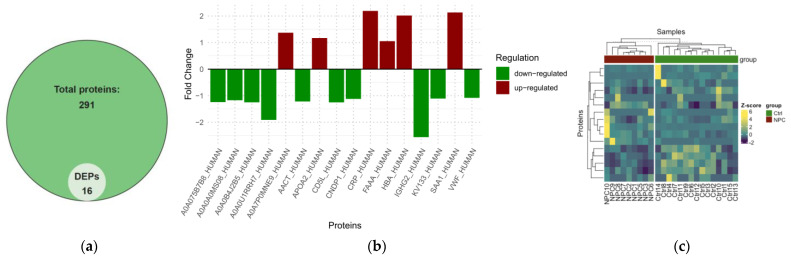
Visualization of identified proteins and DEPs between patients and controls: (**a**) Venn diagram representing the total identified proteins and DEPs. (**b**) Bar plot representing the upregulated and downregulated proteins. (**c**) Clustering of DEP proteins alone stratifies patients.

First, we examined the differentially expressed proteins between NPC patients and healthy controls. Under this condition, 16 DEPs were identified from the total number of proteins (Supplementary Material Table S4). This included six upregulated proteins with a fold change ≥1 and 10 downregulated proteins with a fold change ≤1, as presented in the Volcano plot, Venn diagram, Bar plot, and heatmap (Figure 4a and Figure 5a–c). In patients with early versus advanced stages of NPC, only complement C3 (CO3) and sex hormone-binding globulin (SHBG) differentially expressed proteins were observed, both being downregulated, as presented in the volcano plot (Figure 4b). The differential expression analysis was also conducted to unveil the differentiated proteins amongst the identified NPC clusters. This revealed four differentially expressed proteins, including two upregulated and two downregulated proteins. Vitamin D-binding protein (VTDB) and polymeric immunoglobulin receptor (PIGR) proteins were differentially expressed between clusters 1 and 2, while Mannan-binding lectin Serine Protease 2 (MASP2) and Apolipoprotein B-100 (APOB) were identified as DEPs in cluster 1 versus 3 and cluster 2 versus 3, respectively (Figure 4c).

The ten most significant DEPs between patients with NPC and healthy controls were Immunoglobulin heavy constant gamma 2 (IGHG2), Beta-Ala-His dipeptidase (CNDP1), Fumarylacetoacetase (FAH), Alpha-1-antichymotrypsin (AACT), also known as SERPINA3, CD5 antigen-like (CD5L), Apolipoprotein A-II (APOA2), Immunoglobulin heavy variable 3/OR16-12 (IGHV3OR16-12), Serum amyloid A-1 protein (SSA1), C-reactive protein (CRP), and Immunoglobulin heavy constant gamma 1 (IGHG1) (Table 1 and Supplementary Material Table S4).

To assess the diagnostic potential of the DEPs, we performed ROC analysis and calculated the AUC for each protein. Notably, several DEPs showed high values indicating strong discriminative performance, including Histone H2A (AUC = 0.91), IGHG2 (AUC = 0.86), Serine Protease 3 (AUC = 0.85), CD5L (AUC = 0.82), CNDP1 (AUC = 0.79), and IGHV3OR16-12 (AUC = 0.79) (Supplementary File S2).

On the other hand, we conducted biostatistical analyses on the significant DEPs to examine their prognostic significance in NPC. Among these DEPs, the expression of IGHG2 and SERPINA3 showed statistical significance in the early and late stages of NPC. SERPINA3’s expression was also significant across all N stages, particularly between N0 and Nx, and between N1 and Nx. Similarly, SAA1’s expression demonstrated statistical significance across N stages in N0–Nx, N1–Nx, N2–Nx, and N3–Nx (Supplementary File S3).

### 2.3. Pathway Analysis of NPC

**Figure 6:**
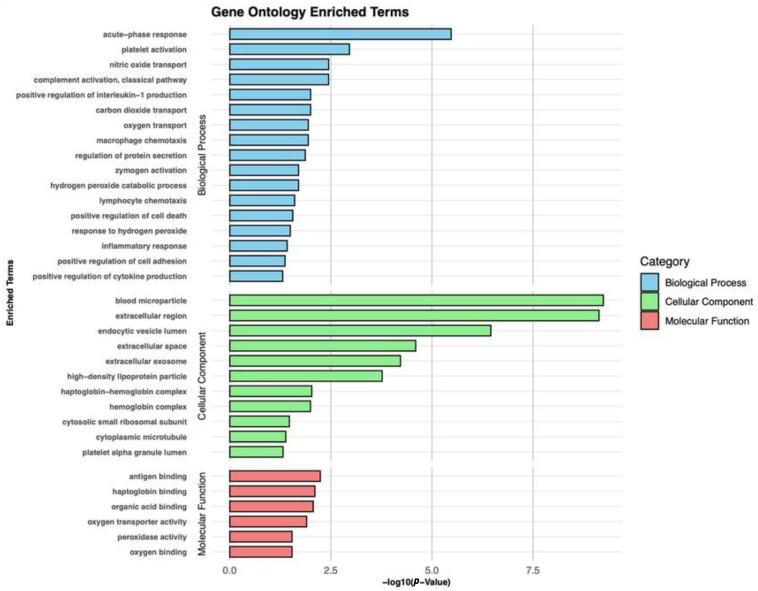
GO pathway analysis in patients with NPC versus healthy controls. Presentation of the significantly enriched terms of BP, CC, and MF.

The Biological Process (BP), Cellular Component (CC), and Molecular Function (MF) categories were analyzed to determine the GO-enriched pathways of the DEPs observed in the plasma proteomic analysis, as seen in Figure 6. The main enriched BP terms in patients with NPC compared to healthy controls were involved in “acute-phase response”, “platelet activation”, “nitric oxide transport”, “complement activation, classical pathway”, and “positive regulation of interleukin-1 production”. DEPs in CC were mainly related to “blood microparticle”, “extracellular region”, “endocytic vesicle lumer”, “extracellular space”, and “extracellular exosome”. Regarding MF, the most significant terms were associated with “antigen-binding”, “haptoglobin binding”, “organic acid binding”, “oxygen transporter activity”, and “peroxidase activity”. Regarding the DEPs identified between NPC stages and between NPC clusters, the pathways analysis using the DAVID bioinformatics tool did not show any enriched terms for GO and KEGG pathways.

### 2.4. Overlapped DEPs Between Tissue and Plasma Samples

**Figure 7:**
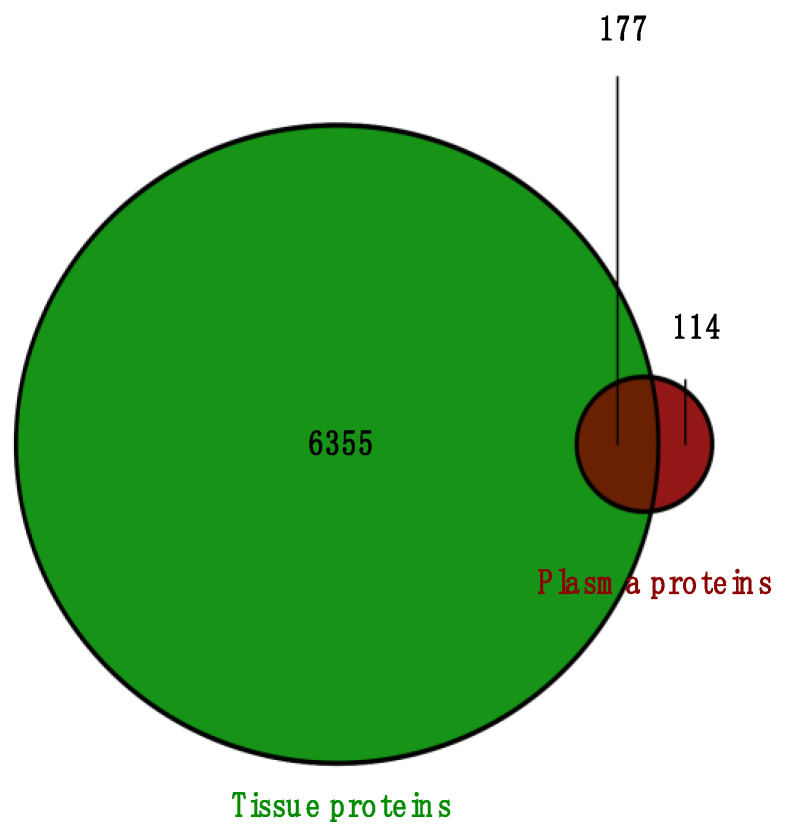
Venn diagram representing the overlapped proteins between the total number of proteins identified in tissue and plasma samples.

Next, we examined the proteins identified in plasma versus the ones identified in tissue samples from our previous study for a more comprehensive analysis. In total, 177 proteins were overlapped between the two sample types of NPC and healthy individuals. A Venn diagram was generated to illustrate the total number of proteins identified (Figure 7). When analyzing the differentially expressed proteins from both studies, we found that five DEPs were identified in both tissues and plasma, as presented in Table 2. SERPINA3 and APOA2 were overlapped between identified DEPs in plasma NPC versus controls and DEPs identified in tissue NPC versus controls, as well as tissue NPC clusters. IGHG2 was common between plasma and tissues in the NPC versus controls condition, and the tissue NPC male versus female condition. Finally, FAH and Hemoglobin subunit alpha (HBA) were overlapped between plasma and tissue NPC versus controls.

## 3. Discussion

We applied mass spectrometry-based shotgun (bottom-up) proteomics with label-free quantification by using a data-dependent acquisition method on plasma samples for the first time within the Moroccan population and throughout the African continent. Bioinformatics analysis of proteomics data was conducted on 200 protein fractions from 25 samples of plasma, including 10 samples of patients with NPC and 15 samples of healthy controls. In total, 291 proteins and 2702 unique peptides were identified, of which 16 proteins were differentially expressed between patients with NPC and healthy individuals. These DEPs were used in the pathways analysis to examine their biological significance in NPC. The main enriched terms of biological processes were related to the immune response and inflammation. The acute-phase response, identified as the primary enriched term, represents a systemic reaction to inflammation, infection, and tissue repair. In the context of chronic inflammation, long-term activation of the acute-phase response could create a tumor-promoting environment [10]. This reaction is characterized by elevated levels of acute-phase proteins triggered by increased production of specific cytokines, including interleukin-1 [11].

The positive regulation of interleukin-1 production was another significantly enriched term of BP, a pro-inflammatory cytokine that is vital in the context of inflammation and immune response [11]. This process may be linked to tumor development and progression by shaping the tumor microenvironment. IL-1 has been reported to promote tumor growth by inducing the expression of inflammatory mediators, including IL-6 and COX2, which support cell survival and proliferation. It also stimulates the production of pro-inflammatory cytokines, chemokines, and growth factors and upregulates stemness-associated genes, such as Bmi1 [12]. Moreover, IL-1 signaling has been implicated in activating the MAPK and NF-κB pathways, which are closely associated with carcinogenesis [13]. Zhi-Hui Yang et al. (2011) investigated the association between IL-1 and nasopharyngeal carcinoma and reported that IL-1A polymorphism may contribute to a risk of developing NPC by affecting the IL-1 serum levels [14]. In another study, Huang YT and colleagues (2010) found EBV LMP1 to upregulate the production of IL1 alpha and beta in NPC, which may contribute to tumor growth [15]. Conversely, IL-1 has also been shown to inhibit tumor growth by enhancing anti-tumor immunity [12].

Another interesting BP term was the activation of platelets, which also contribute to cancer progression. Evidence has shown the implication of platelets in all stages of carcinogenesis, including tumor growth and metastasis [16]. A study by Bailey S and colleagues (2017) identified thrombocytosis, or elevated platelet levels, as a predictor for cancer [17]. In this study, a functional annotation clustering combining BP, CC, and MF of the GO and KEGG pathways revealed a cluster associated with the immune response, further confirming our findings above. This could suggest an interaction of the immune cells with cancer or inflammation that might be caused by NPC.

To assess the diagnostic ability of the differentially expressed proteins (DEPs) observed between patients with NPC and healthy controls, we conducted ROC curve analysis for each protein. We note that given the cohort size, these candidate biomarkers should be treated as extremely preliminary and that further studies on orthogonal cohorts will be required. Several DEPs, including H2A, IGHG2, PRSS3, and CD5L, showed high AUC values, indicating potential clinical utility of these DEPs as candidate plasma biomarkers for NPC. Histone H2A, one of the core components of the nucleosome that plays a critical role in compacting DNA, gene expression regulation, and DNA binding and repair, showed excellent discrimination in ROC analysis. H2A variants like H2AX, H2AZ, and H2A1 were previously reported to be altered and implicated in several cancer types, including sarcoma, breast, colorectal, brain tumor, and head and neck [18]. Notably, H2A modifications, such as acetylation or methylation, were found to play a role in tumor suppressor gene silencing or oncogene activation [19]. Studies have also positioned H2A as a biomarker candidate and therapeutic target for cancer. For example, Rasmussen et al. reported that the nucleosome H2AZ variant, along with other markers detected in serum, could be crucial for the early detection of cancers like colorectal cancer [20]. We believe the H2A canonical protein was identified for the first time as a differentially expressed protein in NPC plasma and might serve as a significant diagnostic biomarker for this type of cancer.

Immunoglobulin heavy constant gamma 2 (IGHG2), part of the constant region of Immunoglobulin heavy chains, was another canonical DEP between NPC patients and healthy individuals that showed good discrimination. IGHG2 is involved in antibody production and adaptive immunity, a major enriched pathway in this study. Here, this protein has been identified for the first time as a potential circular biomarker for NPC. It is worth noting that IGHG2 also demonstrated prognostic significance in this study and was recognized as differentially expressed in the NPC tumor proteome study. In other cancer types, IGHG2 has been reported, particularly in carcinomas, such as head and neck squamous cell carcinoma, oral squamous cell carcinoma, and cervical carcinomas, as well as others like glioblastoma, although they are mainly related to prognosis [21,22,23,24].

Serine Protease 3 (PRSS3), an overexpressed canonical protein within the trypsin family, also showed good discrimination and potential diagnostic potential in NPC. PRSS3 has been found to be implicated in the progression and metastasis of several cancer types. Ma et al. reported an association of PRSS3 with tumor progression in epithelial ovarian cancer, while Wang and colleagues showed a correlation of PRSS3 with metastasis and poor prognosis in patients with gastric cancer [25,26]. The high expression of PRSS3 was also identified in breast cancer tissues and pancreatic cancer [27,28]. However, PRSS3 identification in plasma or serum as a circulating biomarker is very limited. In this research, PRSS3 was detected as differentially expressed for the first time in cancer plasma, but additional research is warranted to explore the potential of PRSS3 in non-invasive cancer diagnostics. CD5L, also known as an apoptosis inhibitor of macrophage (AIM), is a secreted glycoprotein that plays a significant role in regulating immune responses and inflammation. Proteomic analyses have identified altered CD5L levels from inflammation to cancer, including hepatocellular carcinoma and lung adenocarcinoma [29]. Choi and colleagues observed high levels of CD5L in extracellular vesicles of lung cancer that correlated with those in cancer tissues, suggesting a potential role in the non-invasive diagnosis of lung cancer [30]. In this study, CD5l showed significant potential as a circular diagnostic biomarker for NPC. Further validation of these potential diagnostic biomarkers in independent, larger cohorts is warranted.

To evaluate the prognostic significance of the most significant DEPs identified in NPC cases versus controls, we analyzed their expression across nasopharyngeal carcinoma (NPC) stage categories (early versus late/advanced) and TNM classification. Both IGHG2 and SERPINA3 showed statistically significant differences in expression between the early and late stages of NPC, with SERPINA3 also exhibiting significant variation across all N stages. Serine Protease Inhibitor A3 (SERPINA3) is another canonical protein that plays a crucial role in both the acute-phase response and inflammatory processes (Protein Atlas). We also examined the DEPs identified in plasma that overlapped with those from our FFPE tissue study [31]. IGHG2 was found to be downregulated in plasma between NPC patients and healthy controls but upregulated in tissue samples between NPC cases and controls, as well as between male and female patients. Similarly, SERPINA3 was downregulated in plasma between NPC cases and controls but upregulated in FFPE tissues between NPC cases and healthy controls as well as among defined NPC clusters. As one of the major acute-phase proteins produced by the liver, SERPINA3 is stimulated by TNF-α and interleukins, including IL-1 and IL-6, during inflammation [32]. Research has highlighted the involvement of SERPINA3 in various cancers. For instance, Zhang et al. (2021) reported that the overexpression of SERPINA3 promotes tumor invasion, migration, and epithelial–mesenchymal transition (EMT) in triple-negative breast cancer cells [33]. In another study, Yang et al. (2014) demonstrated that SERPINA3 drives endometrial cancer cell proliferation by regulating the G2/M cell cycle checkpoint, inhibiting apoptosis, and interacting with PI3K/AKT and MAPK/ERK1/2 signaling pathways [34]. In nasopharyngeal cancer, circSERPINA3, a circular RNA form of the protein, was found to promote NPC cell proliferation and invasion, suggesting its oncogenic role in NPC progression [35]. Our findings, aligned with the existing literature, position SERPINA3 as a potential prognostic biomarker and therapeutic target in NPC and other cancers, despite it not having been previously identified in the plasma or serum of NPC.

Serum amyloid A-1 (SAA1), another interesting canonical differentially expressed protein (DEP), was found to be statistically significant across the N stages of NPC in this study. This acute-phase protein is a key player in the body’s inflammatory response, which is also linked to cancer progression. High levels of SAA1 expression have been reported in various cancers, including lung cancer [36], where its overexpression was linked to tumor development. In pancreatic cancer, SAA1 has been shown to promote progression through its involvement in inflammation [37]. Additionally, in ovarian cancer, SAA1 has been implicated as a potential biomarker for metastasis [38], further highlighting its significance in cancer biology. Although research on SAA1 in NPC is limited, key findings suggest its potential role in tumor progression. Lung et al. (2015) [39] identified SAA1 expression and polymorphisms as being associated with both tumor-suppressive activities and a higher risk of NPC. Moreover, Li et al. (2020) found that SAA1, along with TNM staging and EBV DNA levels, could serve as independent prognostic factors for NPC, underscoring its potential as a promising prognostic biomarker for the disease [39,40].

Although C-reactive protein (CRP) expression was not statistically significant across NPC staging, this differentially expressed protein, implicated in the acute-phase response and other pathways, including the inflammatory response, was of interest. The blood levels of this key acute-phase protein have served as a marker to detect active inflammatory responses, including those associated with cancer. CRP has been associated with various cancer types, including lung, breast, colorectal, and head and neck cancers, in numerous studies. These findings further reinforce the link between chronic inflammation and tumor development [41]. A notable study investigating the role of CRP in overall cancer and site-specific cancers found that a higher concentration of CRP was linked to an increased risk of developing overall cancer, with positive linear associations observed in esophagus and stomach cancers [42]. The study also reported patterns of non-linear associations, including a fast-to-low increase in head and neck cancers, suggesting CRP as a potential biomarker for cancer. CRP has also been implicated in nasopharyngeal carcinoma (NPC). Studies have shown that high levels of CRP in the blood are associated with poorer survival outcomes, including both baseline CRP levels and CRP kinetics [43,44]. Furthermore, the CRP/Albumin ratio has been explored as an inflammation-based prognostic factor in NPC. Y Zhang et al. (2016) found that patients with high CRP/Alb ratios had significantly worse overall survival, highlighting the ratio’s potential as a valuable prognostic indicator [45]. Additionally, a meta-analysis by S Yang et al. (2019) confirmed CRP as a key prognostic marker in NPC [46]. Our findings, which demonstrate overexpression of CRP in NPC plasma samples compared to healthy controls, further confirm its potential role as a biomarker for this type of cancer. In addition to EBV DNA, the differentially expressed proteins identified in our study could serve as valuable blood markers for the diagnosis and prognosis of NPC.

Similarly to our NPC cancer tissue study [31], and because of the late declaration problem of NPC, we aimed to examine the identified differentially expressed proteins in plasma samples between patients with early and advanced stages of NPC. In this study, 80% of patients had an advanced stage at diagnosis. The analysis revealed two differentially expressed proteins between early and advanced stages, including complement C3 (C3) and sex hormone-binding globulin (SHBG), both being downregulated proteins. Investigating these proteins in UniProt and Human Plasma PeptideAtlas showed that complement C3, a canonical protein, was implicated in several biological processes, including inflammatory response, immune response, and positive regulation of angiogenesis. C3 is a central player of the complement system, and its downregulation might affect the body’s ability to provide an effective immune response against tumor cells, which could suggest a role in carcinogenesis by affecting key reactions in immune surveillance, tumor suppression, and inflammation control. Regarding immune surveillance, the C3 protein was found to play a crucial role in helping the immune system identify cancer cells by regulating the membrane attack complex and several other processes [47]. Its reduced expression might weaken the immune system’s inflammatory response and allow cancer cells to escape immune cells. C3 and other complement proteins were reported to play roles in chronic inflammation, immunosuppression, and angiogenesis [48]. Complement C3 shows potential as a biomarker for early diagnosis; however, further research is required to validate its association with cancer, particularly nasopharyngeal carcinoma. The sex hormone-binding globulin protein, regulating the bioavailability of hormones like testosterone and estrogen, was found to be implicated in various types of cancers–particularly hormone-related cancers such as prostate and breast cancers–particularly when it is downregulated [49,50]. In this study, SHBG was identified for the first time as a downregulated, differentially expressed protein in early versus advanced stages of nasopharyngeal carcinoma (NPC). The role of SHBG in NPC remains unclear and largely unexplored, as NPC is not typically considered a hormone-driven cancer. However, it is important to note that sex hormones may influence NPC progression or susceptibility, especially given the pronounced sex disparity, with NPC being more common in men than women. Further research is needed to investigate the impact of sex hormones on NPC and to explore whether SHBG could serve as a potential marker for early diagnosis.

In this study, we characterized three clusters in the cancerous samples using the highest silhouette score. Among these subtypes, 4 DEPs were identified, including Vitamin D-binding protein (VTDB), polymeric immunoglobulin receptor (PIGR) proteins between clusters 1 and 2, and Mannan-binding lectin Serine Protease 2 (MASP2) and Apolipoprotein B-100 (APOB) between clusters 1 and 3 and clusters 2 and 3, respectively. In our dataset, the PIGR canonical protein, a key component of the mucosal immune system, was found to be downregulated. This finding aligns with the study by Qi et al. (2016), which reported reduced serum levels of PIGR in nasopharyngeal carcinoma (NPC) patients and observed a significantly shorter overall survival in those with downregulated PIGR [51]. Additionally, the study highlighted markedly lower PIGR expression in patients with advanced stages of NPC. Similarly, Y Chang et al. (2005) also reported PIGR downregulation in NPC [52]. In other cancers, Ocak et al. (2012) reported a loss in PIGR expression in lung cancer and its potential role in promoting cell proliferation [53]. Similarly, Fristedt et al. (2014) observed significant downregulation in PIGR and its association with shorter survival in patients with pancreatic and periampullary cancer [54]. Interestingly, high PIGR expression has also been implicated in several cancer types. Despite these findings, there are still controversies regarding the relationship between PIGR and clinical outcomes in cancer [55]. Nevertheless, PIGR remains a promising potential biomarker for the diagnosis and prognosis of NPC. MASP2, a differentially expressed protein, was found to be overexpressed in our study across NPC plasma subtypes. This canonical protein, known for its role in complement activation and immune response, has been associated with cervical cancer stage and metastasis [56]. Additionally, a proteomics study by J Li et al. (2023) reported MASP2 upregulation in patients with thymoma [57]. Given the lack of research on MASP2 in NPC, further studies are needed to explore its potential as a biomarker for cancer and NPC, specifically.

Besides IGHG2 and SERPINA3, other DEPs were identified in both plasma and tissue samples from our previous study [31]. Apolipoprotein A2 (APOA2) was overexpressed in plasma between NPC cases and controls, tissues between NPC cases and controls, and between NPC subtypes. This protein was previously reported as a plasma biomarker for early pancreatic cancer and colorectal cancer detection [58]. It was also used in combination with other markers in the detection of ovarian cancer [59]. A proteomic study by P Baichan et al. (2023) on gallbladder cancer patients of African ancestry also found APOA2 dysregulation in both tissue and plasma, suggesting its potential use as a biomarker for NPC [60]. To the best of our knowledge, APOA2 has been identified as one of the most significant DEPs in nasopharyngeal carcinoma (NPC) for the first time, underscoring its potential as a novel biomarker for this complex disease. Other proteins identified in both plasma and tissues, including FAH and HBA, were upregulated between NPC cases and controls. The overlapped DEPs between the two sample types indicate that plasma proteomics can identify certain tumor-associated signals while also reflecting systemic responses or circulating biomarkers. This suggests a pathway for developing minimally invasive techniques. The distinct differentially expressed proteins (DEPs) found in each sample type further emphasize the complementary role of plasma and tumor proteomics in providing a comprehensive characterization of the disease.

As mentioned before, the majority of molecular studies on nasopharyngeal carcinoma were conducted on the Asian population, most of which were on NPC tumors.

While our findings offer valuable insights into the proteomic profile of NPC, certain limitations should be acknowledged. The cohort of 25 patients is notably small. The collection of high-quality clinical cohorts with associated clinical variables is nontrivial in underserved African communities. Consequently, due to the limited sample size, we were unable to conduct additional analyses to examine all potential differences between DEPs under other conditions and their biological significance in NPC. Additionally, the silhouette score of the clustering analysis was relatively low, though it still provided valuable insights. We also recognize the 291 proteins identified in plasma as a limitation, as recent studies with Bruker TIMS-TOF (Billerica, MA, USA) or Thermo ASTRAL instruments (San Jose, CA, USA) are able to achieve much wider coverage, particularly when using DIA workflows. However, it is important to note that the data were collected and analyzed in 2021–2022 using an LTQ-Orbitrap-Elite. In future research, we hope to analyze these samples and larger, orthogonal cohorts using one of the newer measurement methods that may allow us to quantify a greater number of proteins. Lastly, affinity reagents were not available for the majority of the identified differentially abundant proteins. Consequently, follow-up using immunoassay (e.g., Western and ELISA) was not tractable. Future directions include performing a more comprehensive analysis with a larger cohort to validate our results for biomarker discovery. Furthermore, we aim to complement our proteomics studies with a whole-exome genomics analysis of NPC in Moroccan patients to achieve a more complete characterization of the molecular profile of this complex disease.

## 4. Materials and Methods

### 4.1. Sample Collection

Blood samples were collected after obtaining informed consent. This included ten samples from patients diagnosed with NPC between 2016 and 2020 at the Oncology Clinic of Tangier and fifteen samples from healthy individuals collected at the Medical Laboratory of Tangier. Whole-blood specimens were obtained in 2 5 mL EDTA tubes by a specialized nurse, having, in the end, 50 blood tubes. Epidemiological and clinical data of patients with NPC were collected from medical records and organized into a database. All patients diagnosed with NPC were confirmed to have the undifferentiated non-keratinizing histological subtype of this cancer type, with a majority being male (90%). The median and mean ages were 41.5 and 42.6 years old, respectively. Approximately 80% of patients were diagnosed at a late stage (III, IVa-IVb) based on the TNM classification from the AJCC/UICC 8th edition. The control group had a median and mean age of 32 and 38.4 years old, respectively, with 53% being males. This research study was authorized by the Biomedical Research Ethics Committee (CERB 86-22) and all patients’ and controls’ confidentiality was ensured. Additionally, it was conducted following the Declaration of Helsinki.

### 4.2. Sample Preparation

After collection, blood samples were transferred to the lab on ice. Each EDTA blood tube from each sample (a total of 25 tubes) was directly centrifuged for 10 to 20 min at 1300× *g* for plasma separation at room temperature after gently inverting the tubes (8 to 10 times). The plasma was carefully collected and placed into a microcentrifuge tube without disturbing the buffy coat. Three aliquots were then stored at −80 °C after labeling and securing the vials.

After being transferred to Stanford, plasma samples were depleted using the Pierce Albumin/IgG Removal Kit from Thermo Scientific (Rockford, IL, USA), removing human serum/plasma albumin (HSA) and IgG classes that comprise more than 70% of total plasma protein before protein digestion. The protocol was followed without deviation as described [61]. Following depletion, proteins were quantified using the Micro-BCA assay kit from Thermo. Electrophoresis, using NuPAGE Bis-Tris Mini Gels from Thermo Fisher Scientific, was used to ensure the samples were not degraded during processing.

Depleted plasma was moved from each sample to a fresh tube for 50 ug of protein while on ice. This was followed by the addition of 80 µL of 100 mM ABC and 200 mM Tris(2-carboxyethyl) phosphine (TCEP) reducing agent for a final concentration of 10 mM, followed by a 1 h incubation at 65 °C. Tryptic digestion was performed as described [62]. After digestion, the Pierce High pH Reversed-Phase Peptide Fractionation Kit was used to fractionate peptide samples with eight distinct elution solutions as described [63]. The samples were subsequently air-dried in a speed-vac for 3 h, reconstituted in 0.1% formic acid in LC-MS water, and placed into autosampler vials for LC-MS analysis.

### 4.3. LC-MS/MS Analysis

The tryptic peptides derived from plasma samples were examined using LCMS/MS by inserting 4 µL of peptides into a 10 µL loop on a Dionex Ultimate Rapid Separation Liquid Chromatography System from Thermo Fisher. The next step involved applying tryptic peptides to a C18 trap column at a designated flow rate of 5 µL/min for a duration of 10 min. The peptides were separated using a phased liquid chromatography gradient consisting of mobile phase A (0.1% formic acid in water) and mobile phase B (0.1% formic acid in acetonitrile) on an analytical column of a 25 cm packed with ReproSilPur 120 C18-AQ resin (Dr. Maisch GmbH, Ammerbuch-Entringen, B-W, Germany). The eluted peptides underwent MS/MS analysis on an LTQ-Orbitrap Elite mass spectrometer from Thermo Fisher Scientific (Waltham, Altham, MA, USA). The mobile phase B was kept at 2% for the first 10 min of the gradient program and ramped up to 35% B over the next 100 min. After that, it rose to 85% B over 2 min and held for 7 min at a steady flow rate of 0.4 µL/min. The mass spectrometry program consisted of a mass resolution of 60,000 by selecting the top 10 most abundant ions from each MS1 scan within the 400–1800 *m*/*z* range. The abundant ions were subject to higher energy collision-induced dissociation with a normalized collision energy of 35 eV in a data-dependent mode, with dynamic exclusion activated for 30 s.

### 4.4. Bioinformatics and Data Analysis

#### 4.4.1. Protein Identification and Quantification

The resulting raw data from LC-MS/MS analysis was searched using MaxQuant (MQ) version 2.4.2.0, conducted using the integrated Andromeda search engine with the UniProt-SwissProt Human Proteome database as a reference. The default parameters were employed using the label-free quantification (LFQ) method [64]. Group 0 represented the 10 NPC samples, whereas Group 1 represented the 15 control samples.

Using the LFQ intensity of the identified proteins in NPC cases, we conducted an unsupervised clustering analysis to identify interesting groups or patterns within our NPC cohort without any defined labels. This was carried out in the R programming language using K-means clustering to determine the distinct clusters, based on the protein expression intensities and silhouette analysis, to validate the consistency of the K-clustering results by choosing the highest silhouette score. Packages such as factoextra version 1.0.7 and cluster version 2.1.4 were utilized in R to generate the silhouette plots for this analysis.

#### 4.4.2. Differential Expression Analysis

The MSstats package in R (version 4.6.5) was utilized to perform the differential expression analysis of the data-dependent acquisition (DDA) data [65]. Output files from MQ, including protein group and evidence files, as well as annotation tables, were used as input. The differentially expressed proteins (DEPs) were examined under various conditions, including NPC-defined subtypes (clusters 1 versus 2, 1 versus 3, and 2 versus 3), between patients with NPC and healthy controls, and between patients with early and advanced stages of NPC. In short, data formatting and elimination of contaminants were performed using the MaxQtoMSstatsFormat function in MSstats. Next, the dataProcess function was applied to execute the logarithmic transformation, conduct quantile normalization, and an imputation using Tukey’s median polish (TMP) as the default method. Differential analysis was conducted afterward using the groupComparison function with a significance threshold of *p* < 0.05. By default, MSstats includes multiple hypothesis testing using the Benjamini–Hochberg (BH) procedure to control the False Discovery Rate (FDR). Lastly, heatmaps, Venn diagrams, volcano plots, and other visualizations were generated using different R packages such as ComplexHeatmap version 2.14.0, VennDiagram version 1.7.3, and ggplot 2 version 3.4.4.

Receiver Operating Characteristic (ROC) analysis was conducted to examine the diagnostic potential of the identified DEPs using the pROC package in R. This analysis evaluates the performance and diagnostic ability of candidate biomarkers between two classes: NPC cases and controls. ROC curve plots were generated in R, and the Area Under the ROC curve (AUC) for each protein was calculated. Higher AUC values indicate a better performance of the potential diagnostic biomarker.

#### 4.4.3. Pathway Analysis

Pathway analysis, including Gene Ontology (GO) and Kyoto Encyclopedia of Genes and Genomes (KEGG), for all samples was performed using the DAVID bioinformatics tool, also known as Database for Annotation, Visualization, and Integrated Discovery [66]. The functional annotation of GO was conducted to examine the enriched terms related to Biological Process (BP), Cellular Component (CC), and Molecular Function (MF). Meanwhile, KEGG was employed to identify enriched biological pathways using the EASE (Expression Analysis Systematic Explorer) test with a *p*-value threshold of 0.05. EASE determines the significantly enriched terms in the submitted list of DEPs using a modified Fisher’s exact test.

Functional annotation cluster analysis was also performed to identify different clusters related to the involved DEPs and significantly enriched functions and pathways. The cutoff for statistical significance was established at *p* ≤ 0.05. Visualizations for significantly enriched KEGG and GO pathways were generated using the ggplot 2 R package.

### 4.5. Statistical Analysis

Statistical analysis was performed in R to examine the prognostic significance of the most significant DEPs by looking at protein expression using LFQ intensities across clinicopathological characteristics of NPC, such as staging. To do so, we used the T-test statistical method to compare the expression of proteins between patients with early and late stages of NPC and the ANOVA test to examine DEP significance across different Tumor (T) and Nodal (N) stages. Tukey’s test, a post hoc analysis, was utilized when ANOVA was significant to determine which group means differed. The cutoff for statistical significance was set to *p* < 0.05. Furthermore, box plots of DEPs were created individually using the ggplot 2 package in R.

## 5. Conclusions

Nasopharyngeal carcinoma remains a unique form of head and neck cancer, with distinct geographical distribution and challenges in early diagnosis. Our research provides valuable insights into the molecular landscape of NPC through a shotgun proteomics analysis of plasma samples. It also marks the first plasma proteomic characterization of this type of cancer in Morocco, a North African endemic region for NPC, and serves as a follow-up to our initial tumor proteomic study.

In this study, we identified several differentially expressed proteins across different comparisons, including H2A, IGHG2, SERPINA3, SAA1, CRP, PIGR, and APOA2, as potential biomarkers for NPC’s diagnosis and prognosis. Pathway analysis of these DEPs revealed significant associations with immune response and inflammation pathways. Notably, we also identified several DEPs, such as H2A, IGHG2, PRSS3, CD5L, MASP2, and APOA2, as potential biomarkers for the first time in NPC. These findings enhance our understanding of the plasma molecular profile of NPC and highlight putative biomarkers with potential clinical utility that could improve early detection and prognosis of this malignancy.

## Figures and Tables

**Table 1:** The 10 most significant DEPs in NPC patients compared to healthy individuals.

DEPs	Log2FC	*p*-Value
IGHG2	−2.5596411	3.27 × 10^−5^
CNDP1	−1.1208327	0.0011
FAH	1.05426614	0.0012
SERPINA3	−1.2171742	0.0013
CD5L	−1.2504989	0.0015
APOA2	1.17201774	0.0016
IGHV3OR16-12	−1.2401787	0.0023
SAA1	2.13436008	0.0052
CRP	2.19295647	0.0053
IGHG1	−1.170474	0.0072

**Table 2:** Overlapped DEPs between plasma and tissues under all conditions.

DEPs	Plasma *p*-Value	Plasma Condition	Tissue Condition
IGHG2	3.27 × 10^−5^	NPC vs. controls	NPC vs. controls and NPC male vs. female
SERPINA3	0.0013	NPC vs. controls	NPC vs. controls and NPC clusters
APOA2	0.0016	NPC vs. controls	NPC vs. controls and NPC clusters
FAH	0.0013	NPC vs. controls	NPC vs. controls
HBA	0.0397	NPC vs. controls	NPC vs. controls

## Data Availability

The datasets presented in this article are not readily available as they are part of an ongoing study. However, they will be made accessible upon reasonable request and published in a public repository later on.

## Supplementary Materials

The following supporting information can be downloaded at: https://www.mdpi.com/article/10.3390/ijms26125771/s1.
