# Supplementary material for: Integrated Plasma and Tumor Proteomics of Nasopharyngeal Carcinoma in a Moroccan Cohort
Source: Int J Mol Sci. 2025 Jun 16;26(12):5771. doi: 10.3390/ijms26125771 (PMC12192878; doi:10.3390/ijms26125771)
Supplement: Supplementary file 1 [file ijms-26-05771-s001.zip › Supplementary file S1_diagnosticability.pdf]

Supplementary Material S6

To assess the diagnostic potential of the differentially expressed proteins (DEPs) identified between patients with NPC and healthy controls, we conducted Receiver Operating Characteristic (ROC) analysis and calculated the Area Under the ROC Curve (AUC) for each protein to provide a single numerical summary of the ROC curve. AUC values range from 0 to 1, and the higher the value, the better the performance of the protein between the two groups, indicating its diagnostic potential for nasopharyngeal carcinoma.

In this analysis, several DEPs showed high AUC values, indicating strong discriminative ability:

Histone H2A:

-AUC = 0.91 -> Excellent discrimination

-p-value of DEP = 0.03

-ROC curve:

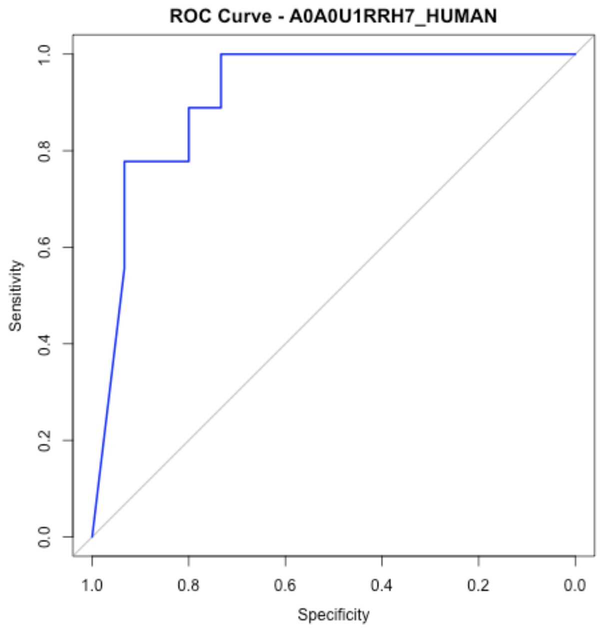

IGHG2:

-AUC = 0.86 -> Good discrimination

-p-value of DEP = 3.27×10^{-5}

-ROC curve:

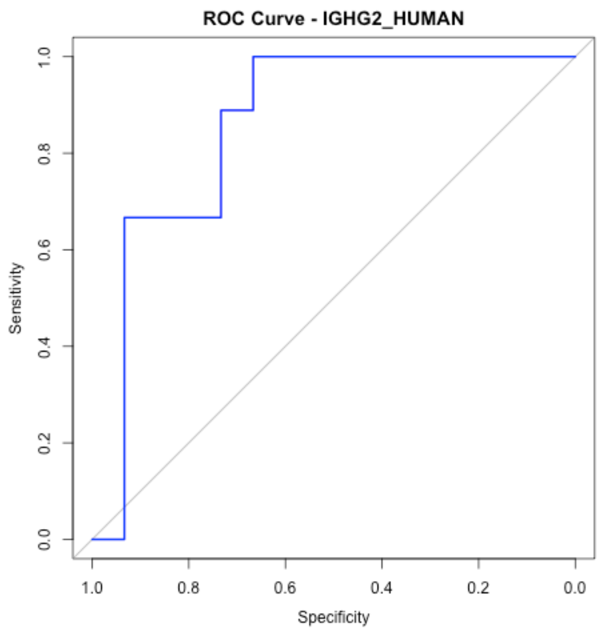

Serine protease 3:

-AUC = 0.85 -> Good discrimination

-p-value of DEP = 0.025

ROC curve:

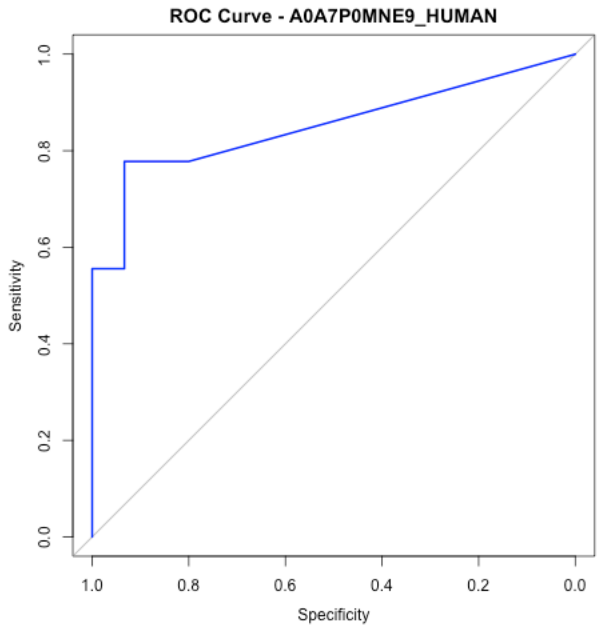

CD5L:

-AUC = 0.82 -> Good discrimination

-p-value of DEP = 0.0015

-ROC curve:

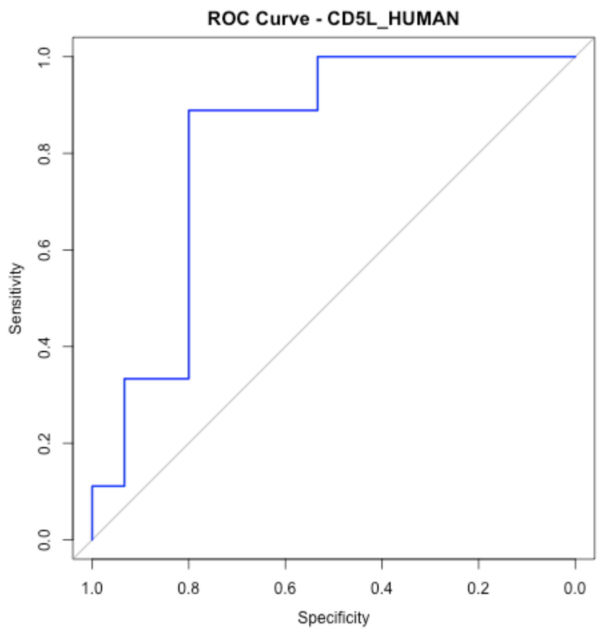

CNDP1:

-AUC = 0.79 -> Fair discrimination

-p-value of DEP = 0.0011

-ROC curve:

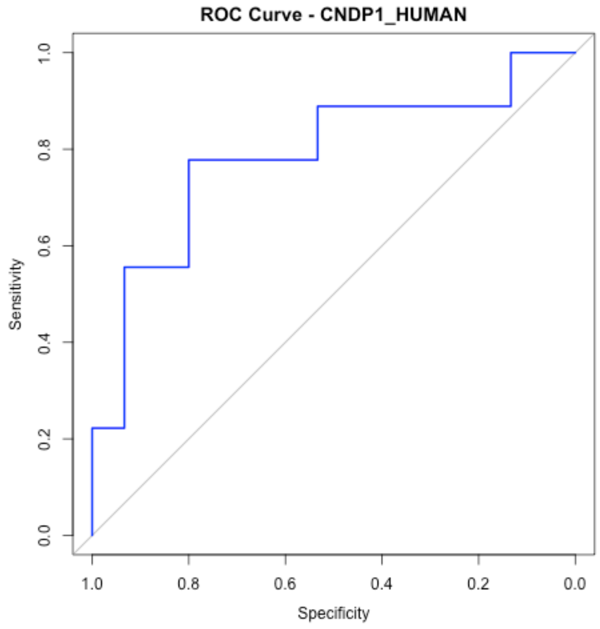

IGHV3OR16-12:

-AUC = 0.79 -> Fair discrimination

-p-value of DEP =

-ROC curve:

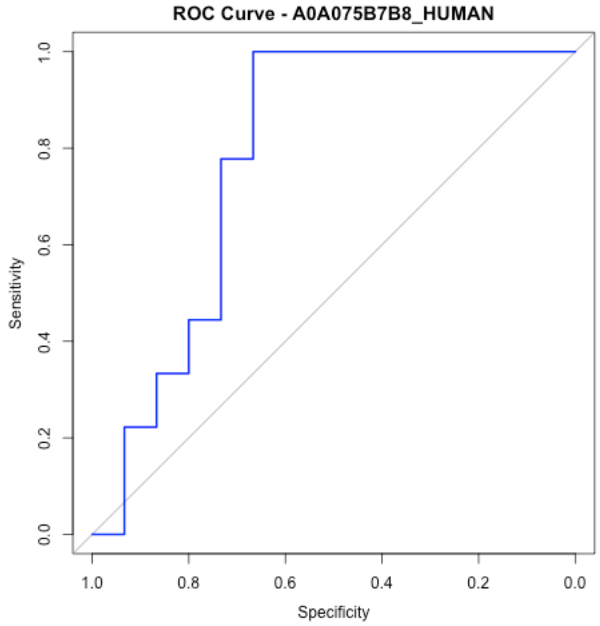

The rest of the differentially expressed proteins:

-Fair discrimination:

IGHG1 (AUC = 0.78)

HBA (AUC = 0.77)

-Borderline/Poor discrimination:

SAA1 (AUC = 0.69)
APOA2 (AUC = 0.68)
KV133 (AUC = 0.58)
SERPINA3 (AUC = 0.53)
CRP (AUC = 0.52)
VWF (AUC = 0.51)
FAH (AUC = 0.50)
IGHV3OR16-9 (AUC = 0.49)
