# Supplementary material for: Integrated Plasma and Tumor Proteomics of Nasopharyngeal Carcinoma in a Moroccan Cohort
Source: Int J Mol Sci. 2025 Jun 16;26(12):5771. doi: 10.3390/ijms26125771 (PMC12192878; doi:10.3390/ijms26125771)
Supplement: Supplementary file 1 [file ijms-26-05771-s001.zip › Supplementary file S2_prognostic_significance.pdf]

Supplementary material S7

The prognostic significance of the 10 most significant DEPs between NPC cases and healthy individuals on clinicopathological characteristics of NPC was examined. The T-test was utilized to compare protein expression in early versus advanced stages of NPC, while the ANOVA test was conducted to assess protein expression significance in T (Tumor progression) and N (Nodal extension) stages. Evaluation of protein expression in the M stage was not included as none of the patients was metastatic at diagnosis. Tukey's test was used to analyze group differences after finding significance in the ANOVA test.

- **Stage categories:**

IGHG2 showed significance in the early and advanced stages of NPC (p-val= 0.024).

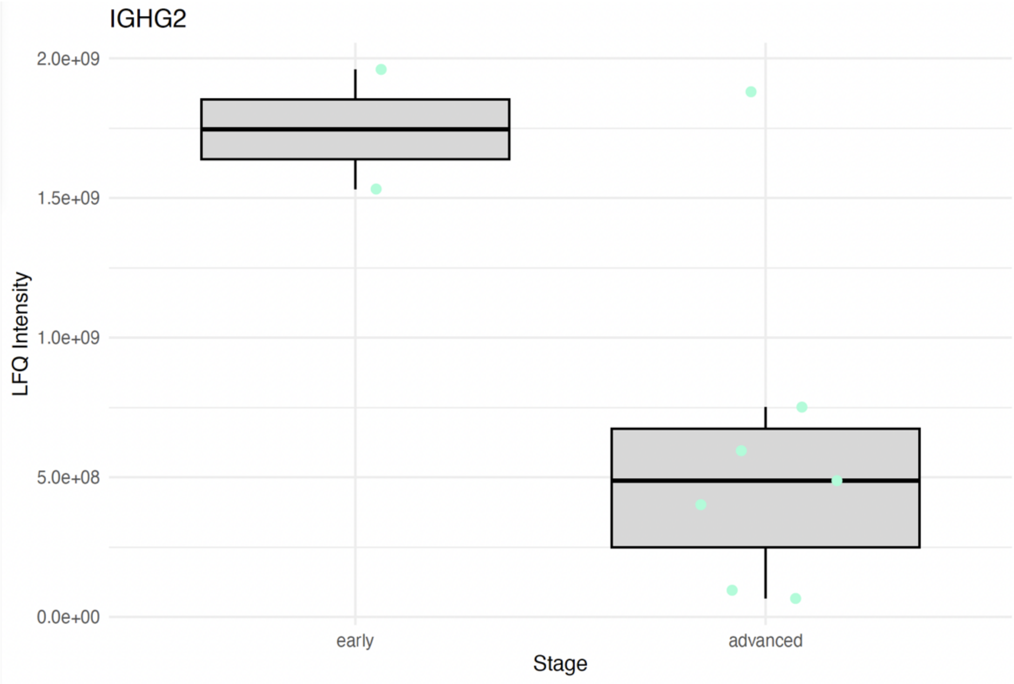


```
        Welch Two Sample t-test

data:  early_stage and late_stage
t = 3.5989, df = 3.8338, p-value = 0.02448
alternative hypothesis: true difference in means is not equal to 0
95 percent confidence interval:
  244265948 2026294337
sample estimates:
 mean of x  mean of y
1746650000  611369857
```

SERPINA3 showed statistical significance in the early and advanced stages of NPC (p-val= 0.032).

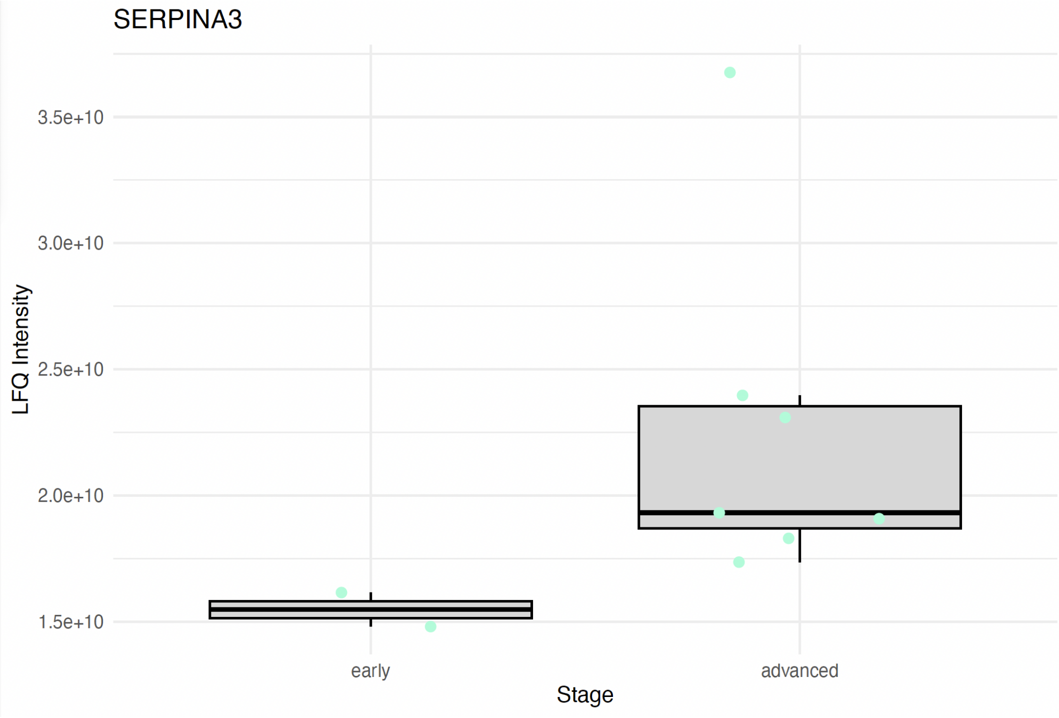


```
        Welch Two Sample t-test

data:  early_stage and late_stage
t = -2.688, df = 6.6745, p-value = 0.03261
alternative hypothesis: true difference in means is not equal to 0
95 percent confidence interval:
 -13359714410   -789142733
sample estimates:
  mean of x   mean of y
15479000000 22553428571
```

- **N Stages:**

SERPINA3 showed statistical significance across N stages of NPC (p-val= 0.045).

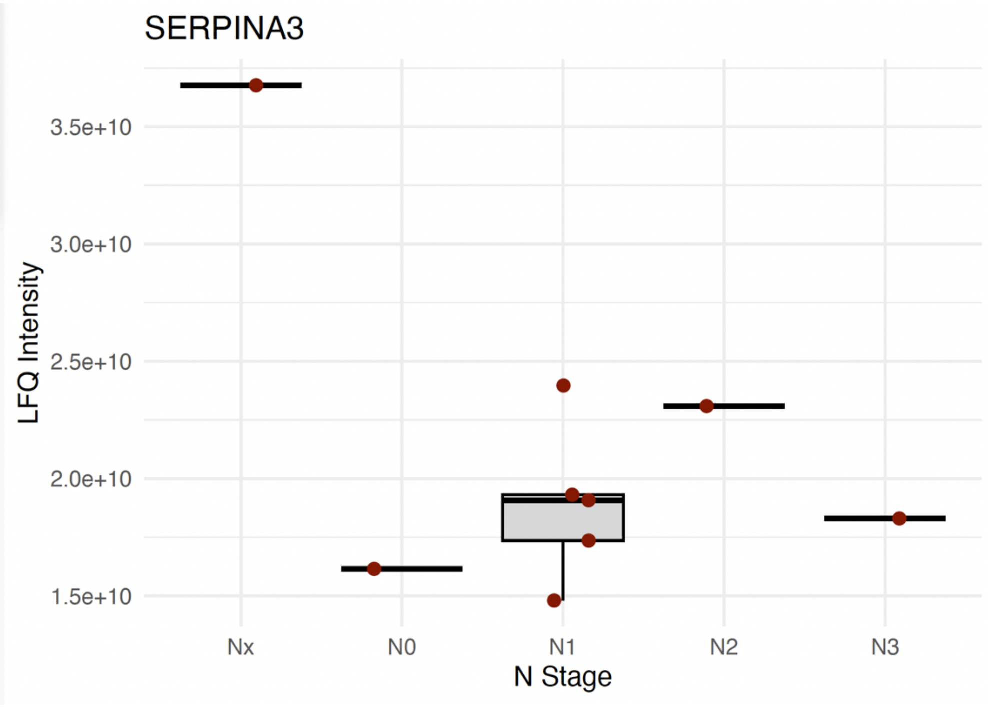

	Df	Sum Sq	Mean Sq	F value	Pr(>F)	
N_Stage	4	3.056e+20	7.639e+19	6.787	0.0452	*
Residuals	4	4.502e+19	1.126e+19			

$N_Stage

	diff	lwr	upr	p adj
N0-Nx	-20610000000	-41702370483	482370483	0.0539129
N1-Nx	-17858200000	-34196279922	-1520120078	0.0372011
N2-Nx	-13672000000	-34764370483	7420370483	0.1797066
N3-Nx	-18462000000	-39554370483	2630370483	0.0764145
N1-N0	2751800000	-13586279922	19089879922	0.9332708
N2-N0	6938000000	-14154370483	28030370483	0.6284511
N3-N0	2148000000	-18944370483	23240370483	0.9881176
N2-N1	4186200000	-12151879922	20524279922	0.7830627
N3-N1	-603800000	-16941879922	15734279922	0.9997585
N3-N2	-4790000000	-25882370483	16302370483	0.8403857

SAA1 showed statistical significance across N stages of NPC (p-val= 0.012).

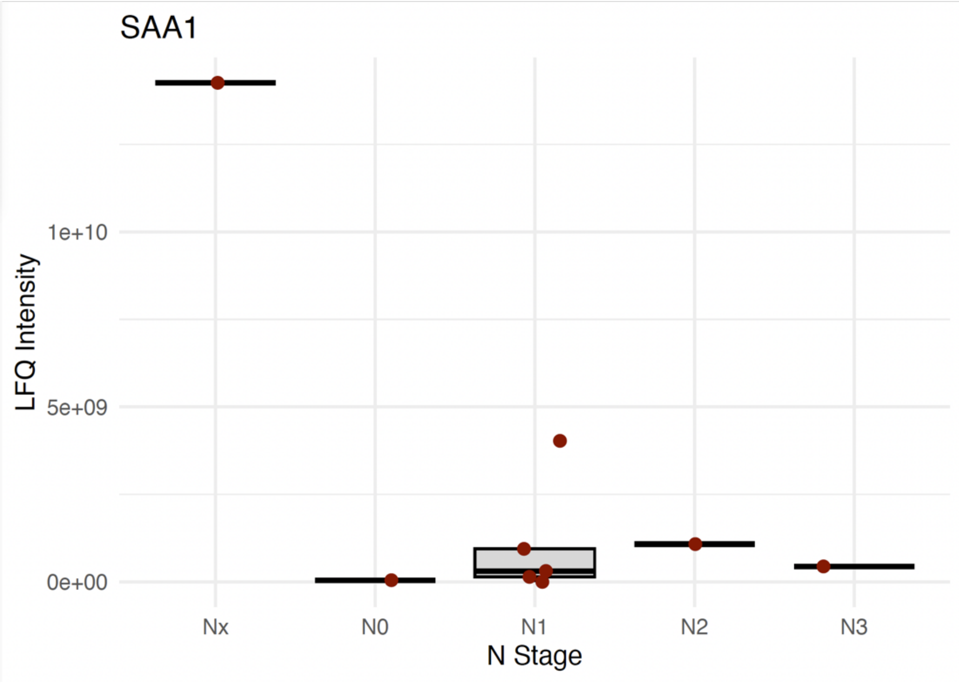

	Df	Sum Sq	Mean Sq	F value	Pr(>F)	
N_Stage	4	1.603e+20	4.009e+19	14.12	0.0125	*
Residuals	4	1.135e+19	2.838e+18			

	diff	lwr	upr	p adj
N0-Nx	-14209196000	-24800600293	-3617791707	0.0182564
N1-Nx	-13172314000	-21376380488	-4968247512	0.0095125
N2-Nx	-13178300000	-23769704293	-2586895707	0.0238233
N3-Nx	-13816310000	-24407714293	-3224905707	0.0201691
N1-N0	1036882000	-7167184488	9240948488	0.9743224
N2-N0	1030896000	-9560508293	11622300293	0.9899316
N3-N0	392886000	-10198518293	10984290293	0.9997549
N2-N1	-5986000	-8210052488	8198080488	1.0000000
N3-N1	-643996000	-8848062488	7560070488	0.9954891
N3-N2	-638010000	-11229414293	9953394293	0.9983637

The two-sample t-test and ANOVA test didn't show statistical significance in the rest of DEPs.
