# Supplementary material for: Integrated Plasma and Tumor Proteomics of Nasopharyngeal Carcinoma in a Moroccan Cohort
Source: Int J Mol Sci. 2025 Jun 16;26(12):5771. doi: 10.3390/ijms26125771 (PMC12192878; doi:10.3390/ijms26125771)
Supplement: Supplementary file 1 [file ijms-26-05771-s001.zip › Supplementary file S3_Data quality metrics.pdf]

Supplemental Material S4

Summary

We employed LC-MS/MS shotgun proteomics with label-free quantification, utilizing a data-dependent acquisition method on 200 fractions derived from 25 plasma samples (8 fractions per sample), including 10 NPC cases and 15 healthy controls. Plasma was initially depleted using the Pierce Albumin/IgG Removal Kit from Thermo Scientific, digested with trypsin, and fractionated using the Pierce High pH Reversed-Phase Peptide Fractionation Kit in 2021-2022. The resulting raw files from the LC-MS/MS analysis were processed using MaxQuant software. Further data processing was performed in R with the MSstats package for differential expression analysis (details in the methods section of the paper). One of the samples didn't show consistent LC-MS results, so only nine NPC samples with 8 fractions each, along with the control samples, were retained for the bioinformatics analysis.

Data Completeness pre-processing:

In this study, 291 proteins without contaminations and reverse proteins and 2702 unique peptides ranging from 7 to 45 amino acids were identified across all samples.

Proteins with LFQ intensity = 0 were considered missing and removed.

The number of proteins and unique peptides identified and quantified in each sample is shown in the following figure.

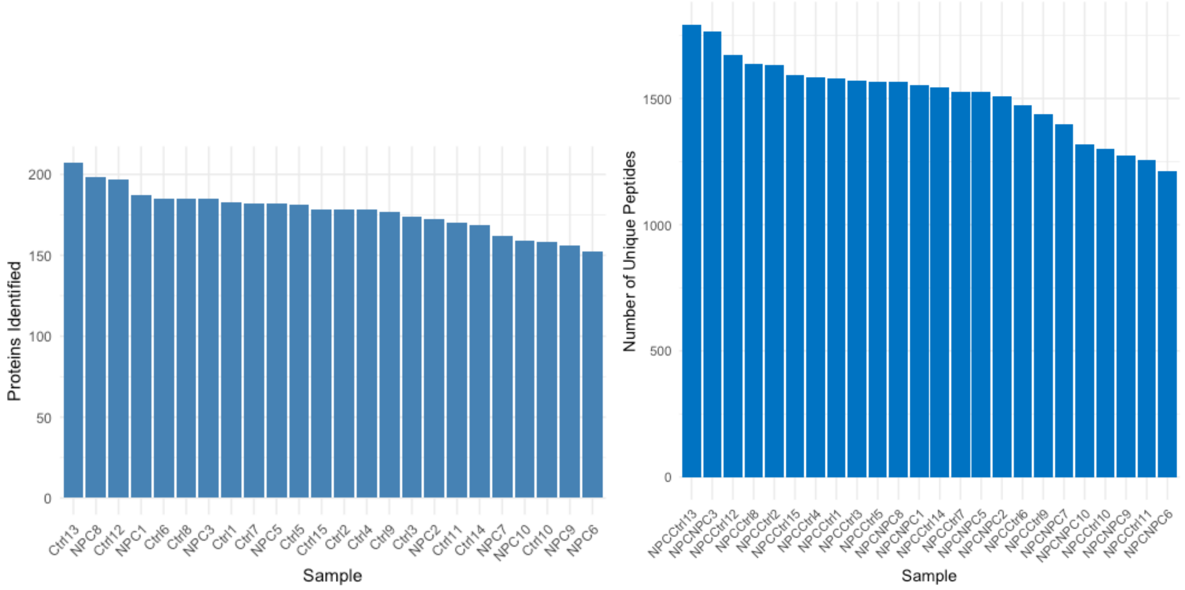

(a) (b)

Figure 1: Barr plot illustrating the number of proteins (a) and unique peptides (b) identified in each sample

A total of 147 proteins were detected in **≥75%** of samples, whereas 117 proteins were detected in **≥90%** of samples. Only 25 proteins had an intensity of 0 in any of the samples.

Additionally, the LFQ matrix exhibited a data completeness of approximately **61%**, reflecting the typical missingness encountered in DDA-based label-free proteomics datasets. This missingness primarily stems from proteins being below the detection threshold or inconsistently quantified across runs. To ensure robustness in downstream analyses, the data were processed using quantile normalization and Tukey's median Polish imputation in R with the MSstats package.

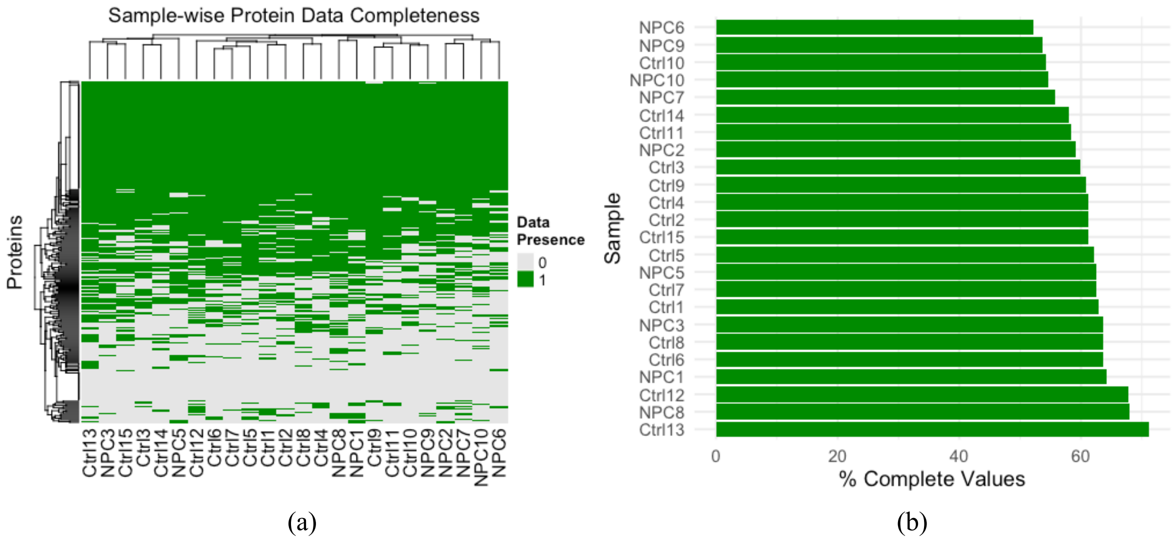

Figure 2: Data completeness visualization: (a). Heatmap showing the presence of proteins's LFQ intensity across samples; (b). Barr plot illustrating the % of complete values across samples

After using the ProcessData function in MSstats, which includes Log2 transformation, normalization, and imputation, the data completeness improved to 71.03%. After filtering out proteins that were present in less than 50% of the samples, the completeness reached 90.95%, as illustrated in the following figure.

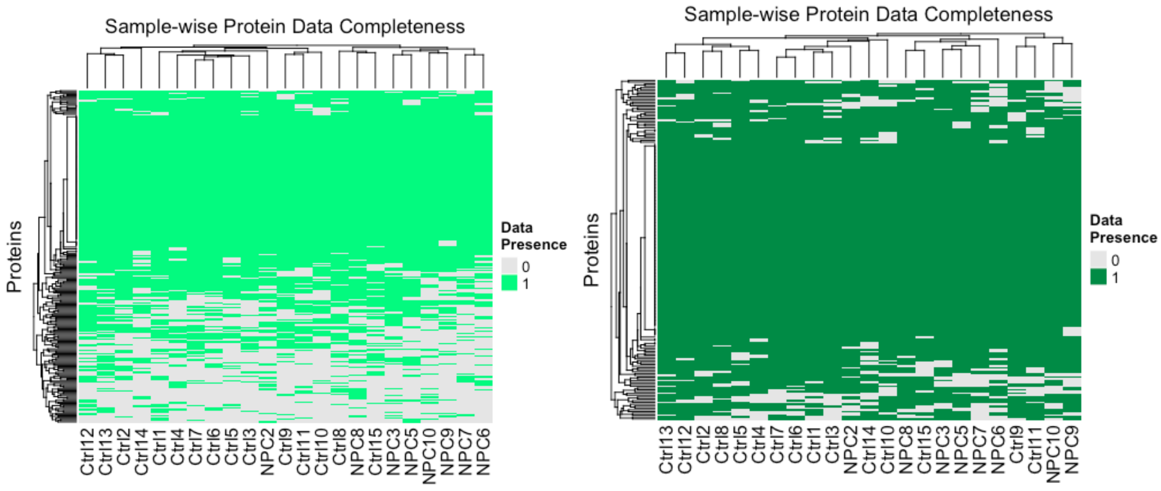

(a) (b)

Figure 3: Heatmaps showing data completeness: (a). Visualization of data completeness after processing without filtering proteins present in less than 50% of the samples; (b). Visualization of data completeness after processing with filtering proteins present in less than 50% of the samples.

Missing values assessment:

We generated a heatmap showing the distribution of proteins with present and missing data across all plasma samples pre-normalization and imputation (Figure 4).

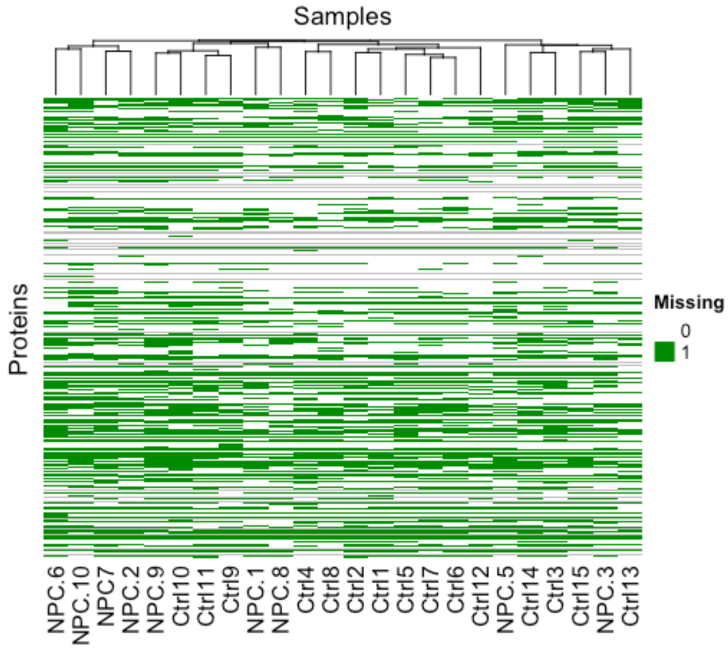

Figure 4: Heatmap showing missing values across plasma samples

Reproducibility and variability

Biological reproducibility from processed Plasma MSstats data

-Sample-to-sample Pearson correlation was computed using MSstats-processed, quantile-normalized protein intensities:

To explore the reproducibility and molecular patterns across plasma samples, we generated a Pearson correlation heatmap based on the expression profiles of proteins across NPC and healthy individuals. The heatmap revealed a stronger degree of similarity among controls and partial clustering among NPC samples, suggesting consistent technical quality along with possible biological heterogeneity in tumors. Moreover, the majority of sample-sample correlations exceeded 0.75, which is favorable and anticipated in LFQ DDA data, although a few samples exhibited lower correlations, likely due to biological variability (Figure 5a).

We also produced a Pearson correlation heatmap for tissue samples (Figure 5b) that were integrated with our plasma proteomics. This revealed two distinct clusters corresponding to NPC and control samples, indicating strong intra-group correlations and distinct inter-group separation. Within each group, samples exhibited high similarity in their protein expression patterns, as shown by the dense red blocks along the diagonal. Conversely, the reduced or negative correlations between NPC and control samples, represented by white to blue areas, highlight substantial differences in their global proteomic landscapes. These clustering patterns suggest a clear molecular distinction between tumor and non-tumor tissues, further supporting the biological relevance of the proteomic profiles captured in our tumor study (see Reffai et al. 2024 for details).

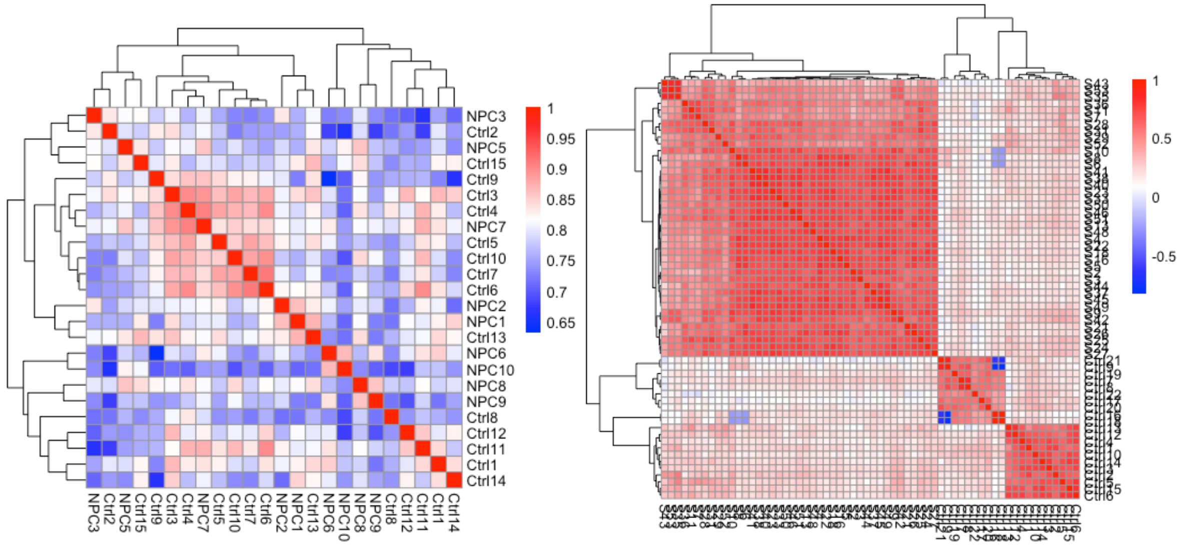

(a) (b)

Figure 5: Heatmap showing Pearson correlation of normalized protein intensities across biological samples (a). plasma samples; (b). tissue samples

-Principal Component Analysis (PCA):

The PCA plot illustrates the distribution of all plasma samples based on the first two principal components (PC1 and PC2), which together account for 20.8% of the total variance, a reasonable proportion for high-dimensional LFQ proteomics data. Each point represents an individual sample, colored by group: control (Ctrl, blue) and nasopharyngeal carcinoma (NPC, red). The analysis demonstrates a separation trend between control and NPC groups along PC1, suggesting distinct global protein expression profiles between the two conditions. Samples are labeled to show individual biological replicates, as shown in Figure 6a. The NPC group shows greater dispersion, reflecting the inherent heterogeneity of tumors.

The Principal Component Analysis (PCA) plot of the tissue samples effectively highlights the variation in protein expression profiles between the control group (Ctrl) and the nasopharyngeal carcinoma (NPC) samples (Group S). The first principal component (PC1), which represents 27.9% of the total variance, clearly differentiates the two groups along the x-axis, indicating a notable difference in global protein expression patterns between NPC tissues and controls. The second principal component (PC2), which explains 6.5% of the variance, contributes less to the separation but still reveals some variability within the two groups, particularly within controls. The tight clustering of the NPC samples suggests a high degree of consistency and reproducibility among tumor replicates, while the more spread-out distribution of control samples along PC2 may reflect underlying biological variability or differences in sample composition (Figure 6b).

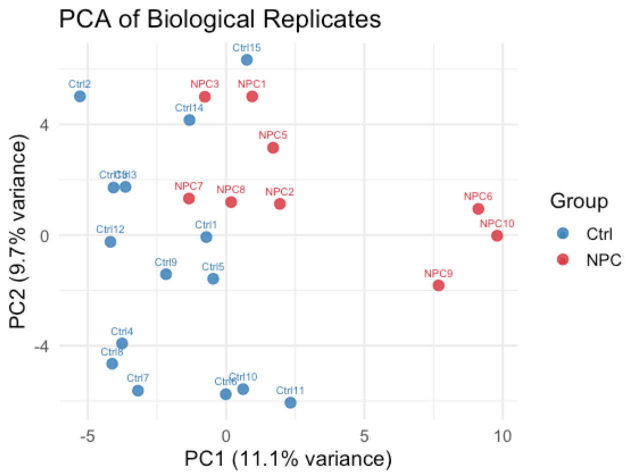

(a)

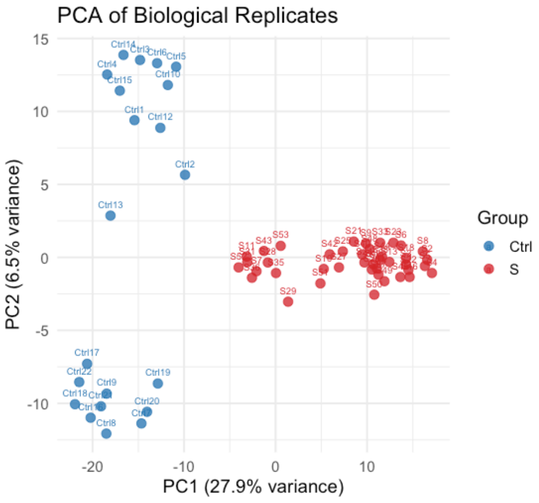

(b)

Figure 6: Principal Component Analysis of biological replicates based on intensity values from (a). NPC and control plasma samples; (b). NPC and control tissue samples

-Coefficient of Variability:

We also computed the coefficient of variation (CV) for each protein identified and quantified in plasma and tissue samples to evaluate the reproducibility and consistency of the proteomic measurements across samples. The CV values were calculated using normalized and log-transformed data as before, and the resulting scatter plot displays the distribution of CVs across all quantified proteins. Most proteins showed low CVs, indicating stable and reproducible quantification, which plasma proteins showing slightly lower CVs compared to tissue proteins.

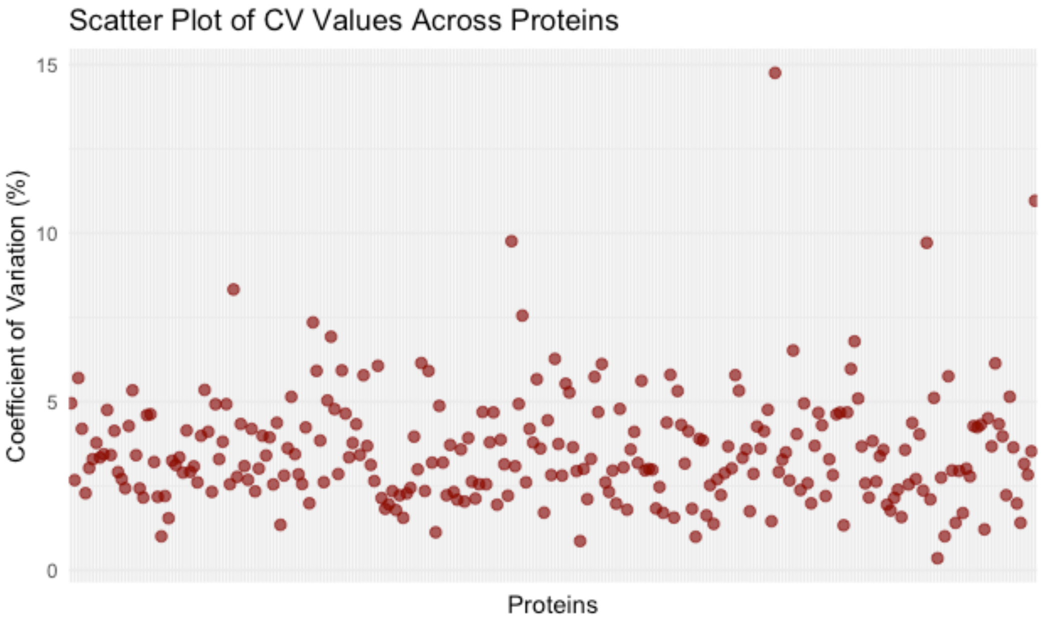

(a)

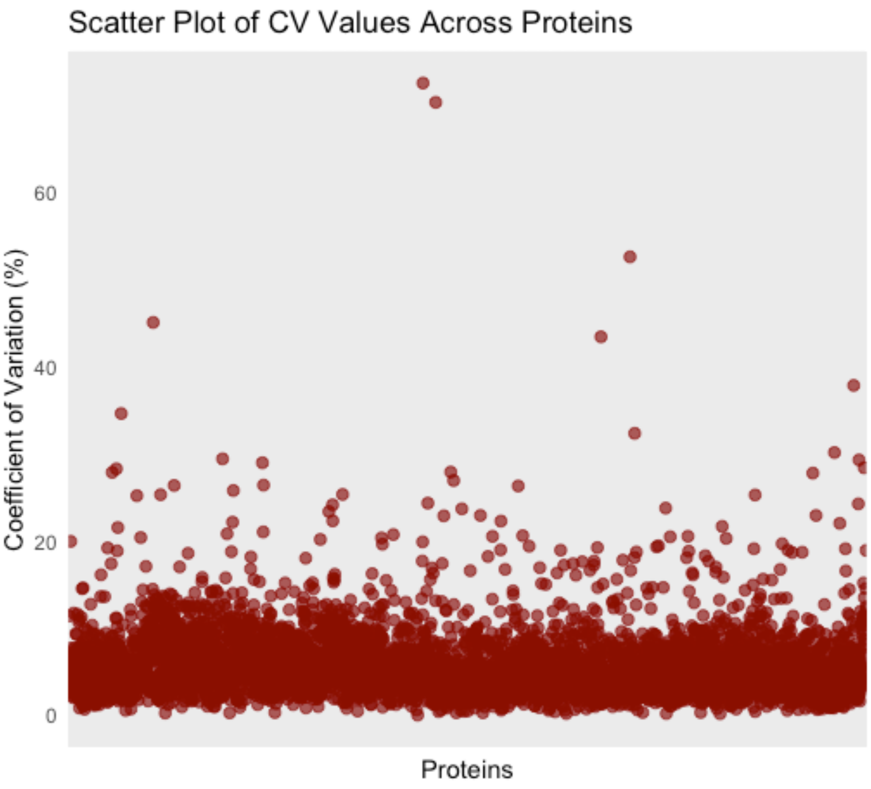

(b)

Figure 7: Scatter plot illustrating the coefficient of variation values for all quantified proteins (a). plasma samples; (b) tissue samples.
